# A Molecular View of Autophagy in Lepidoptera

**DOI:** 10.1155/2014/902315

**Published:** 2014-07-16

**Authors:** Davide Romanelli, Barbara Casati, Eleonora Franzetti, Gianluca Tettamanti

**Affiliations:** Department of Biotechnology and Life Sciences, University of Insubria, Via J. H. Dunant 3, 21100 Varese, Italy

## Abstract

Metamorphosis represents a critical phase in the development of holometabolous insects, during which the larval body is completely reorganized: in fact, most of the larval organs undergo remodeling or completely degenerate before the final structure of the adult insect is rebuilt. In the past, increasing evidence emerged concerning the intervention of autophagy and apoptosis in the cell death processes that occur in larval organs of Lepidoptera during metamorphosis, but a molecular characterization of these pathways was undertaken only in recent years. In addition to developmentally programmed autophagy, there is growing interest in starvation-induced autophagy. Therefore we are now entering a new era of research on autophagy that foreshadows clarification of the role and regulatory mechanisms underlying this self-digesting process in Lepidoptera. Given that some of the most important lepidopteran species of high economic importance, such as the silkworm, *Bombyx mori*, belong to this insect order, we expect that this information on autophagy will be fully exploited not only in basic research but also for practical applications.

## 1. Introduction

More than one million species of insects have been described in the literature and they are characterized by a great morphological and physiological heterogeneity. Despite the great diversity among insects that belong to different orders, metamorphosis, a biological process by which the adult organism is formed through a series of gradual changes, represents a common feature. In this context, holometabolous insects clearly represent an intriguing model because the overall body organization of the larva changes completely during metamorphosis: most of the organs undergo deep remodeling or even completely degenerate at that stage, and proliferation and differentiation processes are required to form the new body structure typical of the adult insect [1].

Over the years it has been demonstrated that larval organs in holometabolous insects degenerate mainly through apoptosis and autophagy. However, while considerable attention has been paid to the role of apoptosis and to characterizing the signals and mediators that regulate this process, the story of autophagy is more complex and still incomplete, probably because clear markers to identify this process in insects were not available for a long time [2].

In this paper, we deal with aspects of autophagy in Lepidoptera, such as identification of autophagic genes and proteins, dissection of the autophagic pathway, and assessment of the relationship between autophagy and apoptosis, and we discuss specific features of the autophagic process in these insects that are expected to be unraveled in the near future.

## 2. A Glimpse of the Past and a Look into the Future

Due to indisputable advantages such as a short and well-known life cycle, well-characterized developmental genetics, and a wide range of molecular tools for gene manipulation,* Drosophila melanogaster* has played the primary role among insects and has traditionally represented a key model system for studies on autophagy (for a complete review see [3]). It also represents a reference point for other insect species, as outlined in the present paper. Although Lepidoptera cannot offer the advantages of a model organism such as* Drosophila* by far, the larvae of these insects are amenable to endocrinological, physiological, and developmental biology studies and, owing to the increasing repertoire of molecular tools for some lepidopteran species, the study of autophagy in these insects has been reappraised in recent years. In addition, Lepidoptera have an added value in terms of practical applications. In fact, some of the most important species of high economic importance, such as the silkworm,* Bombyx mori*, which is bred for silk production, or insect pests that reduce crop production or destroy stored food grains, belong to this insect order. Therefore, a deep understanding of the processes that regulate metamorphosis in the larval organs of these organisms, cell death processes in particular, could provide essential information that could be exploited for practical applications.

Studies on autophagy in Lepidoptera date back to a half-century ago, when Locke and Collins [4, 5] provided evidence that autophagy occurs in the larvae of the larger canna leafroller,* Calpodes ethlius*, during metamorphosis. Since then, dozens of studies have reported morphological or biochemical features that can be ascribed to this self-digesting process, and in the last 15–20 years molecular evidence of autophagy in moths and butterflies emerged.

**Figure 1:**
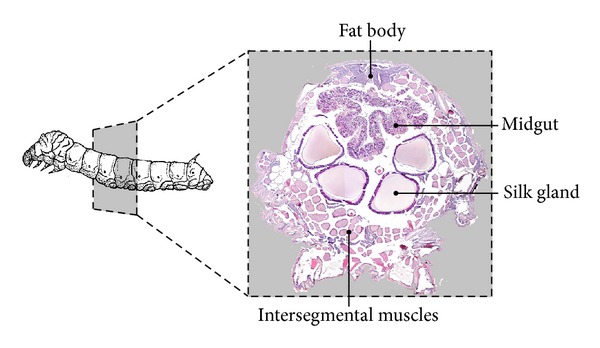
Schematic representation of the main larval organs that undergo programmed autophagy during metamorphosis in* Bombyx mori*.

Basically, three main periods can be identified in Lepidoptera autophagy research: the morphological, the biochemical, and the molecular period. During the early period, the examination of several larval organs, including fat body [6], midgut [7], silk gland [8–10], intersegmental muscles (ISMs) [11, 12], and wing epithelium [13], gave evidence of the presence of autophagic features, such as autophagosomes and lysosomes, during metamorphic degeneration (Figure 1). In particular, ISMs were demonstrated to be a powerful model. Some articles published in the sixties by Lockshin and Williams on ISMs of silk moths [14, 15], not only introduced the term “programmed cell death” in relation to insect tissue development, but also led to the classification of this degenerative process as Type 2 cell death that will be detailed below. Subsequent studies demonstrated that the elimination of the muscle cytoplasm was brought about by a combination of lysosomal and proteasomal activity. In fact, the demise of ISMs is accompanied by increased activity of cathepsins and acid phosphatases, and lysosome-like organelles containing mitochondria could be observed during this process [14, 15]. Moreover, ISMs death is mediated by enhanced protein catabolism via the ubiquitin-proteasome pathway:* de novo* expression of several genes associated to proteolysis is required and all the main components of this pathway are increased [16–18]. Thus ISMs are an excellent system for studying the role of autophagy in muscle atrophy and death, which may also provide useful information for clinical disorders.

Some of the pioneering studies hypothesized that the rough endoplasmic reticulum, Golgi, and mitochondria might be a source for the membrane that formed the autophagosome. Lysosomes are critical for autophagy and several authors directed their attention to this organelle, showing an accumulation of lysosomes in several larval tissues undergoing autophagy and a parallel increase in acid phosphatase levels [6, 9, 11, 19]. Matsuura and colleagues [19] also suggested a model in which acid phosphatase activity occurs in a biphasic pattern during degeneration of the silk gland, which supports a dual role of autophagy during the demise of this organ. The importance of lysosomal enzymes in the degeneration of larval organs reported in these early papers has been definitively demonstrated in two recent studies in the silkworm [20, 21], by cloning cathepsins B and D, two enzymes whose expression is induced by 20-hydroxyecdysone (20E) and that work in a fashion similar to acid phosphatase: in fact, silencing these two genes through RNA interference (RNAi) negatively impacts pupal development.

Alongside this morphological approach to the study of autophagy, a biochemical-physiological approach rapidly evolved. This approach was essentially devoted to identifying the activating and regulatory signals of autophagy in lepidopteran organs and tissues. Evidence obtained in the fat body demonstrated that autophagy is triggered in the larva by a pulse of 20E and the onset of this process can be prevented by applying ligatures behind the brain-ring gland complex [22, 23]. Moreover, experiments performed* in vitro* not only confirmed that this hormone is able to switch on autophagy in fat body cells, but also showed that, once the cells are committed to autophagy and the process is activated, ecdysone is no longer required for completion since autophagy can continue in a hormone-free medium [24].

The new period that has emerged in the last ten years is primarily centered on a molecular view of autophagy and is mainly based on the significant advances that were made after genome sequencing of* B. mori* [25, 26]. In general, the increasing use of microarrays and RNA-Seq has deepened our knowledge of gene expression patterns in multiple tissues or in different conditions such as metamorphosis and immune response, and facilitated the identification of miRNAs [27]. Major results have also been gained from extensive proteomic analyses performed on more than ten different tissues and organs in the silkworm. In addition, several genetic tools with which larvae can be genetically manipulated are now available for* B. mori*. Silkworm is, in fact, amenable to systemic RNAi and stable germline transformation, thus providing an opportunity to gain insight into the function of autophagic proteins through gene knockdown or overexpression. As an example, the expression of several autophagy-related (*ATG*) genes has been efficiently silenced in the fat body of this lepidopteran [28].

Additional effort has been invested in identifying and characterizing genes involved in the autophagic process and in evaluating their expression in larval tissues of different Lepidoptera. From the results obtained in these studies, and in particular thanks to the bioinformatics analyses performed by Zhang and colleagues [29], it became rapidly clear that homologs of most of the genes that belong to the autophagic pathway are present in the silkworm genome. These include multiple* ATG* genes (Table 1), originally identified in yeast and subsequently in higher eukaryotes, and genes involved in the phosphatidylinositol-3-kinase (PI3K) signal transduction pathway and in the formation of autophagosomes [29, 30].

*ATG* genes constituted the primary target of these investigations. They were identified in silkworm starting from yeast, other insects (mainly* Drosophila*), and human sequences available in public databases [29, 31]. Expression of* ATG* genes has been described in different silkworm tissues. In particular,* BmATG1*,* BmATG5*,* BmATG6*, and* BmATG8* were expressed in peritracheal athrocytes and gonads [31, 32]. A striking upregulation of various* ATG* genes during fifth larval instar and metamorphosis of this insect has also been observed in the larval midgut epithelium [32, 33], silk gland [29, 34, 35], and fat body [28, 32]. In fat body it has also been demonstrated that 20E levels affect the expression of these genes* in vivo*, confirming that the ecdysone titer in the hemolymph is linked to the induction of the autophagic program in the larval organs [28]. It is important to underscore that, although monitoring of* ATG* gene transcription is not recommended as a general readout for autophagy [36], the results obtained by using several markers to monitor activation of autophagy in these tissues confirm that, at least in midgut, silk gland, and fat body of* B. mori*, these changes in gene expression just prior to cell death of these organs correlate with autophagic activity.

Among the* ATG* genes,* ATG1* and* ATG8* are particularly interesting because they play a pivotal role in the autophagic process.* ATG1* is necessary and sufficient itself to induce the autophagic process in* Drosophila*: overexpression of this gene triggers downstream pathways and stimulates autophagy in a kinase-dependent manner [37]. In silkworm,* BmATG1* expression is significantly enhanced during the first day of the spinning phase both in the larval fat body and midgut and can be quickly induced in the fat body by complete food withdrawal [32] and 20E injection [28].* BmATG1* cloning revealed the expression of two full-length coding sequences (*BmATG1* transcript variants A and B), closely related to orthologs of other insects [32]. An ecdysone response element (EcRE) is located within the* BmATG1* promoter, confirming its role as a 20E primary-response gene [28]. The encoded BmAtg1 proteins share extensive homology with orthologs from yeast to mammals, showing high conservation at the N-terminal region where the catalytic domain and ATP- and Mg-binding sites are located, as revealed by sequence analysis and* in silico* prediction of its three-dimensional structure [32]. On the other hand, Atg8 is a key factor in autophagosome formation and can be used as an undisputable marker for autophagy given its localization on the autophagosome membrane [38].* BmATG8* expression peaks in several silkworm tissues at the onset of metamorphosis as well as after injecting 20E [28, 29, 33]. Determination of the crystal structure of BmAtg8 showed that not only the sequence but also structural domains such as the ubiquitin fold and some essential amino acid residues are conserved [39]. Recently, Zhang et al. [40], by expressing Atg8 protein fused with different tags in* Spodoptera litura* cells, demonstrated that Atg8 has both a nuclear and cytoplasmic localization when expressed at high levels and that the protein moves to the cytoplasm when autophagy is activated. This study not only provides evidence on localization and shuttling of Atg8 between cytoplasm and nucleus, but also gives interesting information for the interpretation of autophagic assays based on Atg8-fusion proteins.

The evidence collected from the aforementioned proteomic and molecular studies set the stage for functional analyses of the molecular pathways and signals that regulate autophagy in Lepidoptera.

## 3. Developmentally Programmed Autophagy: A Lesson from* Drosophila*?

Programmed cell death (PCD) plays an important role in animals in the removal of superfluous or damaged cells and is thus a key process that sculpts tissues and organs during morphogenesis. Three major forms of cell death have been described based on morphological criteria. Type 1 PCD (apoptosis) shows nuclear condensation and fragmentation and membrane blebbing and formation of apoptotic bodies that are engulfed by phagocytes and depends on caspase activation. Type 2 PCD (autophagic cell death) is characterized by an accumulation of autophagosomes and autolysosomes in the cytoplasm that self-digest the cell and thus it is less dependent on phagocytes that clear up cellular debris. Type 3 PCD (necrotic cell death) involves cell swelling, membrane rupture, and release of cytoplasmic content in the extracellular environment [41]. The occurrence of autophagic cell death has been postulated in different organisms, but since these descriptions are mainly based on morphological features, only in limited cases autophagy has been shown to have a causative role in cell death. For this reason, it is possible to outline two main scenarios: (i) “cell death by autophagy,” where autophagy actively contributes to the cell death process, and inhibiting autophagy rescues the cell from the death stimulus, keeping it alive; and (ii) “cell death with autophagy,” where autophagy simply accompanies the cell death process and does not have an active role in it [42]. Thus autophagy does not seem to have a universal role in executing PCD but rather is required in a context-specific manner. The demise of larval organs in holometabolous insects, which requires both autophagy and apoptosis, represents an ideal model for tackling such questions: in particular, one of the most intriguing and controversial problems is the understanding of the function of autophagy and its regulatory mechanisms in this context. Studies in* Drosophila* have demonstrated that autophagy actively intervenes during the removal of salivary gland. In fact, mutations in several* ATG* genes or knockdown of* ATG* genes specifically in salivary gland cells are associated with incomplete degradation of this organ during metamorphosis. Moreover, overexpression of* ATG1* in salivary gland induces premature degradation in a caspase-independent manner [43]. Interestingly, the combined inhibition of autophagy and caspases enhances the impairment of the degradation process, thus suggesting that removal of the gland requires a cooperative action of autophagy and caspases [43]. Concerning midgut, an early paper suggested that histolysis of this organ can be inhibited by ectopically expressing the caspase inhibitor p35 [44], and Yin and Thummel showed that midgut deficient in the proapoptotic genes* Rpr* and* Hid* fails to undergo apoptosis during metamorphosis [45]. Interestingly, subsequent work has demonstrated that autophagy plays a role in the PCD of larval midgut, too, although in a distinctive manner. Similarly to salivary gland, loss-of-function* ATG* mutants or knockdown of* ATG1* and* ATG18* severely delays midgut removal [46], and overexpression of* ATG1* is sufficient to induce autophagy and premature removal of midgut cells [47]. In contrast to salivary gland, even though caspases are active, they are not necessary for midgut removal, so that the combined inhibition of autophagy and caspases does not increase the delay of this process compared to inhibition of autophagy alone [46]. In fat body, induction of autophagy by* ATG1* overexpression is sufficient to induce caspase-dependent cell death: in this case, cells show apoptotic features corroborating the hypothesis that autophagy can induce apoptosis [37]. An additional example of a context-dependent relationship between autophagy and apoptosis in the fly comes from oogenesis. Indeed, during oogenesis, cell death requires autophagy and components of the apoptotic machinery: Nezis et al. [48] have demonstrated that the autophagic degradation of the inhibitor of apoptosis protein (IAP) dBruce is required to induce DNA fragmentation, thus postulating a role for autophagy in caspase activation and occurrence of apoptosis. All of the aforementioned studies support the conclusion that the involvement of caspases during developmentally programmed autophagy is tissue-specific in* Drosophila*.

In Lepidoptera, the coexistence of autophagic and apoptotic features has been frequently described in many organs that die during metamorphosis: DNA fragmentation, apoptotic nuclei, and caspase activation have been detected in silk gland [49], fat body [50–53], midgut [33, 54–58], and other tissues [59–62] in which autophagic features have been found as well. Even though most of these studies are based on morphological observations—and functional studies will be necessary to demonstrate a role of autophagy in tissue degradation—this copresence of autophagic and apoptotic characteristics within the same organ does not seem to represent mere redundancy and adds evidence to the hypothesis that there is an overlap in the regulatory pathways of autophagy and apoptosis in Lepidoptera as well.

**Figure 2:**
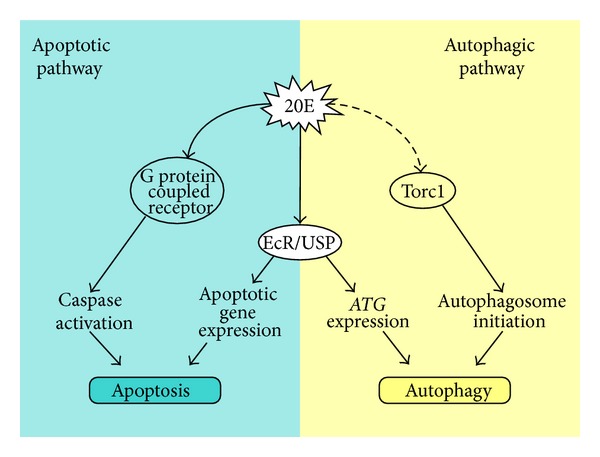
Activation of autophagy and apoptosis by 20E. The model is based on results obtained in the silk gland [64] and fat body [28].

In insects, the intertwining between autophagy and apoptosis extends to the activation phase, which is triggered by 20E [1, 63]. The signaling pathways of these two mechanisms in Lepidoptera have been studied in silk gland and fat body, indicating both peculiarities and overlaps. Although some evidence suggests that apoptosis can be triggered by a single injection of 20E in silkworm fat body [52], Sakurai and coworkers [64–67] showed that, in the anterior silk gland, cell death is regulated by a double pulse of this hormone, which peaks twice during larval-pupal transition. The first peak (commitment peak) is able to activate the so-called genomic response mediated by the EcR/USP receptor complex, upregulating the expression of apoptosis-related genes. Moreover, at the commitment peak, the larvae stop feeding and silk spinning is induced. The second peak, called the metamorphic peak, which occurs during the pupal stage and is higher than the previous one, may activate the so-called nongenomic response via a putative membrane ecdysone receptor, probably a G protein-coupled receptor. This nongenomic response activates the apoptotic machinery and the effector caspase-3-like protease (Figure 2). Interestingly, Tian and colleagues [28] proposed a similar model specifically based on autophagy for a genomic/nongenomic response to 20E. They clearly showed that, in* B. mori* fat body, the increase in 20E titer upregulates most of the* ATG* genes during molting and pupation. By injecting 20E in larvae on the second day of the fifth instar,* ATG* genes are transcriptionally upregulated, target of rapamycin complex 1 (Torc1) is inhibited (as confirmed by a decreased phosphorylation of Eukaryotic translation initiation factor 4E-binding protein 1 (EIF4EBP1)), and autophagic compartments are increased. In contrast, autophagy is reduced by RNAi of* ATG1* and* USP* genes and in* EcR* dominant-negative mutants during larval-pupal transition. All this information produced a working model in which 20E is able to increase autophagy in two different ways: (i) by acting through the receptor complex EcR/USP to activate transcription of* ATG* genes, both directly (this happens for* ATG1* thanks to the presence of an EcRE in the promoter) and indirectly (through the action of Br-C and downstream proteins) and (ii) by inhibiting the PI3K/Torc1 pathway, allowing activation of the downstream Atg1/Atg13 complex and initiating autophagosome formation (Figure 2). This is in line with what has been reported for* Drosophila* fat body, where inhibition of the PI3K/Tor signaling by 20E can activate autophagy [68]. Another similarity with* Drosophila* is that autophagy can also be stimulated by injecting rapamycin, a Tor inhibitor, into feeding larvae [28]. However, the effect elicited in the fat body of the silkworm is weaker than that for treatment with 20E, probably reflecting the inability of this drug to increase the expression level of all critical* ATG* genes [28].

It is now becoming progressively clear that, at least in Lepidoptera, the induction of autophagy and apoptosis by 20E is probably more complex. Indeed, although both autophagy and apoptosis can be activated by 20E, it must be emphasized that, while both 20E commitment and metamorphic peak are required to trigger apoptosis, which produces an increase in apoptosis-related gene expression and activation of the apoptotic pathway, respectively, autophagy might be controlled exclusively by the first 20E peak. In fact, a single pulse of 20E injected in the hemocoel is sufficient to activate the autophagic pathway in silkworm fat body [28]. In addition, during larval-pupal transition, autophagy is activated simultaneously with the 20E commitment peak in silkworm larval midgut [33]. A few more words must be devoted to juvenile hormone (JH), the second player involved in regulating insect development and metamorphosis. The common view appears to be that JH inhibits autophagy prior metamorphosis. Accordingly, in* Manduca sexta* it has been demonstrated that topical administration of JH at early phase of last larval instar inhibits autophagy in the fat body, while treatments at later stages results to be ineffective, thus suggesting that autophagy can be activated only when 20E concentration increases, provided that JH is absent or present in a low amount [22, 69]. In Lepidoptera, there is limited evidence on the signaling pathway that links JH to autophagy: in particular, Guo et al. [70], by investigating the role of the putative JH receptor MET during metamorphosis of* B. mori*, reported that RNAi of this gene had a marked effect on the autophagic response at the early wandering stage.

Current data support at least two different potential scenarios to describe the autophagic process in Lepidoptera: caspase-dependent versus caspase-independent cell death involving autophagy. In fact, the expression of active caspases has been detected in the larval midgut of* Heliothis virescens* [54],* B. mori* [33] and* Spodoptera littoralis* [55] at pupal stage, by using antibodies specific for cleaved caspase-3. Moreover, in larval motoneurons of the tobacco hornworm, in which the death process is accompanied by autophagy, administration of a chemical inhibitor suppresses caspase activity and impairs the completion of the cell death process in these cells [60]. These data seem to be in contrast with fat body, where no evidence of executor caspase activity has been reported [50, 52]. A further level of complexity was added some years ago by a study performed in the labial glands of* M. sexta*, where an increase in lysosomal proteolytic activity was observed during the demise of this organ. According to the authors these enzymes could substitute for caspases and activate the apoptotic cascade, given that no caspase activity was detected [71]. In conclusion, as seen above for* Drosophila*, the involvement of caspases in PCD could be context-dependent in Lepidoptera, too.

Although studies performed in silk gland and fat body, as described above, have shed light on the activating pathways of apoptosis and autophagy during PCD in larval organs, uncertainty exists about the true role of autophagy in this setting. In* B. mori* fat body, 20E induces the expression of both apoptotic and* ATG* genes at the onset of metamorphosis. This event is accompanied by DNA fragmentation, appearance of autophagosomes/autolysosomes and increased lysosomal activity [28, 52]. In this case it has been proposed that autophagy functions to exploit nutrients in this adipose tissue in order to support the growth and differentiation of the new adult structures [28]. In the larval midgut of silkworm, autophagy is activated at wandering stage: Franzetti et al. [33] not only showed a large number of autophagic compartments in the midgut epithelium and a huge increase in lysosomes and acid phosphatase activity, but also detected BmAtg8 processing to BmAtg8-PE in these cells. Autophagy is set in motion once the larva stops feeding: it reduces the protein concentration in midgut tissue and induces a striking increase in ATP levels, to cope with starvation. On the other hand, apoptosis is activated later and promotes the demise of larval midgut cells. Thus, in this model, autophagy and apoptosis appear to have different functions: the former plays a prosurvival role for midgut cells deprived of nutrients, while the latter is strictly related to cell death [33]. Although molecular and functional analyses are in progress to unravel the true nature of the autophagic process in dying midgut and fat body, according to the preliminary evidence collected so far, it seems that apoptosis plays the key role in the disappearance of the larval organ, similar to early evidence reported for* Drosophila* [44, 45].

Given that a hierarchical relationship has been demonstrated between autophagy and apoptosis in* Drosophila* nurse cells [48] and fat body [37], it will be interesting to determine whether such a regulation can be found also in the aforementioned settings for Lepidoptera (i.e., midgut, fat body and other organs) and to verify whether the two processes work through interconnected regulatory pathway. If so, the molecular mediators of such cross-talk would need to be identified. Taking the work done on* Drosophila* as a reference, in our opinion two molecular factors deserve particular attention: (1) IAP: in* Drosophila*, dBruce has been shown to provide a mechanistic link between autophagy and cell death [48]. Interestingly, IAP expression changes during midgut remodeling in several Lepidoptera, a situation where autophagy has been shown to occur [55, 58, 72]. Moreover, IAP transcription increases under starvation and decreases after refeeding in* Galleria mellonella*, thus suggesting a link between IAP and autophagy [58]. (2) Atg1: overexpression of Atg1 in* Drosophila* has been demonstrated to induce autophagy and subsequent caspase-dependent cell death with most of Atg1-overexpressing cells removed within 36 hours. In* B. mori* midgut an increase in Atg1 expression is observed during larval-pupal transition [32], concomitantly with autophagy and preceding apoptosis by two days [33]. Therefore, a possible regulation of apoptotic cell death by Atg1 cannot be excluded in this insect model either, an aspect that surely deserves further investigation.

## 4. A Prosurvival Role for Autophagy during Starvation

Although autophagy has been widely described during metamorphosis in holometabolous insects, it must be underlined that this self-eating process probably represents an adaptation of cells to starvation: in yeast, when the cell is subjected to nutrient deprivation, autophagy is activated to break down part of its reserves in order to stay alive until the situation improves [73]. This primary function of autophagy has been conserved and maintained up to pluricellular organisms and, in several insects, an autophagic response has been observed in cells and tissues deprived of nutrients [74–76].

In* Drosophila*, two seminal papers showed that, in the fat body, autophagy is regulated by the PI3K/Tor signaling pathway in response to ecdysone [68] and nutritional stress [76], thus demonstrating that developmentally and nutritionally triggered autophagy are coordinated. The key mediator is Tor, a negative regulator of autophagy activated by the Class I PI3K signaling pathway that affects phosphorylation and repression of Atg1.

In Lepidoptera, too, besides developmentally programmed autophagy, increasing attention is being devoted to the activation and regulation of autophagy following starvation or in relation to nutritional cues, such as lipid metabolism. Several key genes involved in the insulin and PI3K/Akt signaling pathway have been cloned and characterized in the silkworm [77]. In addition, two paralogous* TOR* genes with high sequence similarity,* BmTOR1* and* BmTOR2*, have been identified by Zhou and colleagues [30]. The genomic analysis revealed that BmTor1 is the ortholog, while BmTor2 is then derived after a duplication event. The two* BmTOR* genes have similar expression patterns and tissue distribution in fat body, midgut, silk glands, and other organs, levels being highest during molting and pupation. Both BmTor isoforms can be transcriptionally regulated by starvation and injection of 20E, although BmTor2 seems to respond better to both of these stimuli [30]. The possibility of modifying Tor pathway activation is very useful because the induction and regulation of starvation-induced autophagy can be investigated in Lepidoptera and previous evidence obtained in larvae subjected to food deprivation can be reappraised [32, 75].

In* G. mellonella*, Khoa and Takeda demonstrated that* GmATG8* transcription is upregulated and the expression of both GmAtg8 and GmAtg8-PE proteins increases in larval midgut after 5 days of starvation. After refeeding the larvae,* GmATG8* transcription and protein expression return to physiological levels [75]. This observation suggests that starvation alone is sufficient to activate autophagy in the absence of 20E stimulation, at least in this experimental model. Additional information on the turnover of Atg8 under starvation conditions comes from a study performed in a* S. litura* cell line [78]. In Sl-HP cells, amino acid deprivation causes a striking change in the expression of the autophagic marker Atg8. Interestingly, although a slight increase in GFP-Atg8 spots is observed 1-2 hours after the beginning of starvation, the total levels of Atg8 and Atg8-PE protein expression decrease. This apparently contradictory result can be explained by an enhanced conversion of Atg8 to Atg8-PE and a consequent acceleration of Atg8-PE degradation in the autolysosome. Accordingly, by treating cells with bafilomycin A1, an autophagosome-lysosome fusion inhibitor, this degradation process is slowed down. Based on these results, the authors suggest that, in Lepidoptera, starvation appears to induce autophagosome-autolysosome maturation rather than autophagosome formation [78].

In silkworm the impact of starvation on the onset of autophagy has also been monitored by evaluating the expression of* BmATG1* in the midgut and fat body. After food deprivation, the expression of this gene is increased in fifth instar larvae and this effect is stronger and faster in the fat body than in the midgut [32]. In fact, at least four days of starvation are needed to significantly increase* BmATG1* expression in the midgut. In contrast, autophagy is induced rapidly in the fat body, probably due to the energy storage function of this organ. This evidence is in accordance with a growing body of evidence suggesting that autophagy also has a role in regulating lipid metabolism and intracellular lipid stores during starvation and in response to 20E in Lepidoptera: (i) in* Drosophila*, Wang and colleagues [79] proposed that Rab32, a member of the Ras GTPase subfamily involved in lipid storage, may execute its function by affecting autophagy, thus supporting the notion that the autophagic process is involved in lipid metabolism. Rab32 is highly conserved among insects and its expression in the moth* Helicoverpa armigera* is induced by 20E in epidermis and midgut during metamorphosis, a developmental stage during which the animal does not feed and must rely only on its energy stocks, thus identifying Rab32 as a possible player in this response [80]; (ii) the transcriptional factor FOXO has been proven to be a key regulator of autophagy both in mammals [81, 82] and* Drosophila* [83]. Hossain et al. [84] found that activation of the transcriptional factor FOXO by 20E promotes lipolysis in the fat body. In particular, they cloned the* B. mori* homolog of FOXO and demonstrated that its expression increased, and protein localization changed from cell membrane to the nucleus during fourth instar molting and larval-pupal transition as well as after exposure to 20E both in cell cultures and* in vivo* [84]. According to these data, an involvement of autophagy in lipid metabolism in the silkworm fat body seems plausible, as also suggested by Tian et al. [28]; (iii) in the IPLB-LdFB cell line, established from* Lymantria dispar* fat body, the administration of oligomycin A can induce a starvation-induced autophagic response, as detailed below. A concomitant shift towards lipid metabolism has been observed in these cells during exposure to oligomycin A, supporting a link between autophagy and lipid metabolism [85].

A large array of cell lines has been derived from the larval organs of* B. mori* and other lepidopterans. Along with the undisputed advantages of experimentation in cell lines, concomitant activation of autophagy and apoptosis as well as of other forms of cell death can be induced in some of them. Thus, this* in vitro* model proves to be a powerful and promising system to specifically dissect the autophagic response to starvation and to evaluate its cytoprotective role and/or its relationship with apoptosis or other forms of cell death [86].

In Sl-1 cells (from* S. litura*), glucose starvation increases the number of autophagic vacuoles. If starvation persists for more than 48 hours, these compartments gradually decrease, while apoptosis-related phenotypes appear [87]. In* S. litura* SL-ZSU-1 cell line, inhibition of starvation-induced autophagy with 3-methyladenine promotes a quick apoptotic response. A similar effect is observed in* B. mori* Bm36 cells, in which inhibition of starvation-induced autophagy by the same drug causes necrotic cell death. These observations can be interpreted by hypothesizing a role for autophagy in preventing the onset of cell death under nutrient deprivation conditions [88].

In the IPLB-LdFB cell line, the effect of nutrient starvation on autophagy was investigated by using oligomycin A, an inhibitor of the mitochondrial ATP synthase [85, 89]. Treatment of cells with this drug rapidly decreases the ATP content within 30 minutes and mimics the condition of nutrient scarcity [89, 90]. In a small percentage of cells, this drug promotes the production of a high quantity of reactive oxygen species and subsequent cell damage, leading to apoptotic, oncotic, and necrotic cell death within 48 hours after the treatment. In contrast, in the majority of the cells, administering oligomycin A induces the onset of autophagy (targeting mainly mitochondria) and actin reorganization [89, 91]. This autophagic response precedes cell death and the authors suggested a correlation between autophagy and cell demise. Thus in this model, autophagy seems to be necessary for the cells to completely recover from ATP depletion and cellular damage, promoting cell protection; however, this action is rapidly overtaken and autophagy becomes associated with cell death mechanisms. Interestingly, a proteomic screening, aimed to identify mediators of this autophagy-mediated response to oligomycin A, found a correlation between the activation of autophagy and a drastic reduction in the levels of imaginal disk growth factor (IDGF)-like protein, a mitogenic factor that regulates growth processes in insects [85]. These data suggest that lack of this prosurvival factor in the cell medium may activate signaling that leads to cell demise, prompting future studies on the relationship between cell survival and developmental cell death that involves autophagy in insects.

## 5. Conclusions and Perspectives

The work performed up to now has led to some general conclusions on the autophagic process in Lepidoptera that can be summarized as follows: (i)* ATG* genes identified so far share evolutionary conservation and have been proven to be essential to correctly initiate and complete autophagy; (ii) the remodeling of most larval organs during metamorphosis requires the intervention of autophagy, which is activated by 20E; (iii) despite the prominent role of developmental autophagy in the remodeling of the larval organs, autophagy can also be induced by starvation, thus supporting the notion that in larval tissues of Lepidoptera this “self-eating” mechanism can act also as a cytoprotective process as seen in other organisms; and (iv) autophagic and apoptotic features coexist within the same organs, suggesting a complex intertwining of these two processes during metamorphosis.

The growing number of publications dealing with autophagy in moths and butterflies has established this field as a promising research area that is progressively attracting the interest of an increasing range of researchers. We can envisage that a detailed molecular and functional characterization of autophagy in these insects may have a direct impact on different areas.

*(1) Knowledge of the Basic Mechanisms of Autophagy*. One of the most important advantages of studying autophagy in lepidopteran larval organs is that this process can be dissected in an articulated biological setting and thus its relation to other forms of cell death [33, 49], to metabolic requirements of the cell [85, 89], and to regeneration events (Franzetti et al., in preparation) can be delineated. In particular, the concomitant presence of autophagy and apoptosis within the same larval organ could help to assess the true role of autophagy in a complex developmental context and to determine in these tissues whether true autophagic cell death exists or if autophagy simply accompanies cell death processes.

*(2) Food and Sustainable Agriculture*. Reduction of pesticide distribution is one of the major objectives in sustainable agriculture and is largely being addressed by adopting the use of environmentally safe products, including biological control agents. To this end it must be considered that: (i) a number of antagonistic associations in insects represent underexploited sources of natural compounds which disrupt larval development, reproduction, and immune response of insect pests, frequently by inducing cell death processes [92]; (ii) the bacterium* Bacillus thuringiensis* produces a variety of entomocidal proteins, safe for vertebrates and almost exclusively active against larval stages of lepidopteran, dipteran, and coleopteran insects, which lead to cytotoxicity and cell death events in the insect tissues [93, 94]; and (iii) there is increasing interest in the use of specific nanomaterials that show pronounced toxic effects on insects (the so-called “nanocides”) [95]. All these nonconventional agents, potentially able to misregulate or induce cell death processes in lepidopteran tissues, could represent safe tools able to disrupt larval development. Thus, a complete view of the different cell death processes, and of autophagic events in particular, that occur in lepidopteran larvae could generate basic information of key importance for opening new frontiers in the field of biological control of pest insects in the postgenomic era.

*(3) Health*. Several reports have implicated autophagy, a major route for the bulk degradation of aberrant cytosolic macromolecules and organelles, in aging control. Accordingly, genetic studies showed that* ATG* genes are involved in lifespan control in nematodes [96] and maintenance of basal expression of* ATG8* in the nervous system of* Drosophila* extended the lifespan by 50%, thus demonstrating that autophagy regulates the rate at which the tissues age [97]. Moreover, an evolutionarily conserved role for autophagy in preventing neurodegeneration has been demonstrated in mice and flies. In these models, in fact, loss of autophagy causes an age-dependent accumulation of ubiquitin-containing inclusion bodies in neurons, which disrupt neural function [98–100]. The interaction of Atg proteins with p62, an ubiquitin-binding scaffold protein that accumulates in ubiquitinated protein inclusions [101, 102], may facilitate the selective degradation of these aggregates by autophagy [103]. In this context, silkworm can surely be established as an interesting model organism through which new insights can be gained on the regulation of autophagy via signals linked to oxidative stress, caloric restriction, and energy availability, thus contributing to a significant pool of information that is useful to understand the relationship between autophagy and aging.

*(4) Sericulture*. The silk gland is the largest tissue in the last instar of the silkworm* B. mori* and begins to degenerate shortly before pupation, once the silk cocoon has been completely spun. This degeneration process is driven both by apoptosis and by autophagy [53]. Recently, Ma and colleagues [104] demonstrated that transgenic silkworms with increased Ras activity show an enlargement of the posterior region of the silk gland; this leads to an increased production of fibroin, one of the two constituents of the silk thread. Since modulation of Ras signaling leads to striking consequences on the cell growth in the silk gland, we can foresee similar biotechnological approaches to antagonize autophagy and apoptosis in this organ that aim to reduce its deterioration, thus increasing its longevity and hopefully improving quantitatively the silk yield.

## Figures and Tables

**Table 1:** List of *ATG* genes and proteins identified in Lepidoptera.

Gene	Nucleotide sequence	Gene expression	Protein expression	Protein structure
*ATG1 *	[29, 31, 32]	[28, 32]		[28, 32]
*ATG2 *	[28]	[28]		[28]
*ATG3 *	[29]	[28]		[28]
*ATG4 *	[29]	[28, 105]		[28]
*ATG5 *	[29, 31]	[28, 31, 33, 34]	[34]	[28]
*ATG6 *	[29, 31]	[28, 31, 33–35, 88]	[78, 106]	[28]
*ATG7 *	[29]	[28]		[28]
*ATG8 *	[29, 31, 75, 78, 106]	[28, 29, 31, 33–35, 75]	[28, 33, 34, 40, 75, 78, 106]	[28, 39, 40]
*ATG9 *	[29]	[28]		[28]
*ATG11 *	[28]	[28]		[28]
*ATG12 *	[29]	[28, 29, 34]		[28]
*ATG13 *	[28]	[28]		[28]
*ATG16 *	[29]			[28]
*ATG18 *	[29]	[28]		[28]
